# Supplementary material for: Single Cell Transcriptomic and Chromatin Profiles Suggest Layer Vb Is the Only Layer With Shared Excitatory Cell Types in the Medial and Lateral Entorhinal Cortex
Source: Front Neural Circuits. 2022 Jan 26;15:806154. doi: 10.3389/fncir.2021.806154 (PMC8826650; doi:10.3389/fncir.2021.806154)
Supplement: Supplementary file 14 [file Data_Sheet_1.ZIP › Deep_vs_Sup_Motifs/homerResults/motif2.similar.html]

motif2

## Information for motif2

C
G
A
T
T
C
A
G
C
T
G
A
G
A
T
C
G
T
C
A
A
C
G
T
A
C
G
T
G
C
A
T
G
T
C
A
T
G
C
A
  
Reverse Opposite:  

A
C
G
T
C
A
G
T
C
G
T
A
C
G
T
A
G
T
C
A
A
C
G
T
C
T
A
G
A
G
C
T
A
G
T
C
C
G
T
A
  

|  |  |
| --- | --- |
| p-value: | 1e-140 |
| log p-value: | -3.236e+02 |
| Information Content per bp: | 1.742 |
| Number of Target Sequences with motif | 3008.0 |
| Percentage of Target Sequences with motif | 32.35% |
| Number of Background Sequences with motif | 6864.0 |
| Percentage of Background Sequences with motif | 21.07% |
| Average Position of motif in Targets | 318.7 +/- 250.9bp |
| Average Position of motif in Background | 317.0 +/- 254.0bp |
| Strand Bias (log2 ratio + to - strand density) | -0.1 |
| Multiplicity (# of sites on avg that occur together) | 1.26 |
| Motif File: | file (matrix) reverse opposite |

### Similar de novo motifs found

|  |  |  |  |  |  |  |  |
| --- | --- | --- | --- | --- | --- | --- | --- |
| Rank | Match Score | Redundant Motif | P-value | log P-value | % of Targets | % of Background | Motif file |
| 1 | 0.606 | T C A G C T G A G C T A A C T G A C G T A C T G G C A T C A T G C T G A C G T A | 1e-121 | -280.232139 | 47.01% | 35.14% | motif file (matrix) |
| 2 | 0.947 | A C G T T A C G C G T A A G T C C G T A A G C T A C G T A C G T | 1e-121 | -279.040380 | 38.13% | 26.94% | motif file (matrix) |
| 3 | 0.899 | C G A T A G T C G C A T C A G T G T C A G C T A T G C A C A G T C A T G A G C T T A G C G C T A | 1e-81 | -187.014532 | 27.25% | 19.08% | motif file (matrix) |
| 4 | 0.850 | A C G T C T A G T C G A G A T C C G T A G C A T A C G T A C G T G A C T A G T C G A C T G C T A | 1e-66 | -153.564133 | 15.77% | 9.98% | motif file (matrix) |
| 5 | 0.613 | C T G A C T G A T C G A T C G A C G T A G C A T A G C T A G C T | 1e-52 | -121.000506 | 57.57% | 49.64% | motif file (matrix) |
| 6 | 0.680 | C T G A A C T G T C G A A T C G C T G A G A C T G C A T A G C T | 1e-48 | -111.671775 | 52.30% | 44.69% | motif file (matrix) |
